# Vegetable intake is associated with lower psychological stress via increased anti-inflammatory responses in pregnant women with chronic diseases

**DOI:** 10.1016/j.bbih.2025.101106

**Published:** 2025-09-16

**Authors:** Rosa S. Wong, Keith T.S. Tung, Patrick Ip

**Affiliations:** a Department of Special Education and Counselling, The Education University of Hong Kong, Hong Kong SAR, China; b Department of Paediatrics and Adolescent Medicine, The University of Hong Kong, Hong Kong SAR, China; c Department of Paediatrics and Adolescent Medicine, Hong Kong Children's Hospital, Hospital Authority, Hong Kong SAR, China

**Keywords:** Pregnant women, Vegetable intake, Anti-inflammatory response, Stress, Chronic disease

## Abstract

Women with chronic diseases are susceptible to inflammation and stress during pregnancy. Dietary factors such as vegetable consumption can influence the level of inflammation markers in the body, which research has shown to be associated with stress levels. This study examined the moderating role of vegetable intake in the association between chronic disease history and stress levels via anti-inflammatory cytokine interleukin-10 (IL-10) in pregnant women. We recruited 239 pregnant women from antenatal clinics and used an electronic food frequency questionnaire to survey their vegetable intake. Plasma samples were collected at enrollment and assayed for IL-10. Perceived stress scale was completed one month after the assessment of dietary intake. The relationships among chronic disease history, vegetable intake, IL-10 levels, and stress levels were explored using moderated mediation analysis. Pregnant women with chronic diseases demonstrated elevated stress levels and decreased IL-10 levels compared to those without chronic conditions. However, a significant interaction was observed between vegetable intake and chronic disease history in modulating IL-10 levels (B = 0.09, p = 0.007). Specifically, consuming vegetables was positively associated with IL-10 levels in women with chronic diseases, while this association was not observed in women without chronic conditions. When consuming high levels of vegetables, women with chronic diseases were found to experience lower stress levels than those without (B = −0.43; Boot SE = 0.28; LLCI = −1.06; ULCI = −0.01). It is crucial for pregnant women with chronic diseases to consume a vegetable-rich diet, which could benefit their mental health by potentially reducing inflammation during pregnancy.

## Highlights

• Vegetable intake has anti-inflammatory effects.
• Vegetable intake moderated the association between chronic disease and IL-10 levels in pregnant women.
• Higher IL-10 levels were associated with lower stress levels.
• High vegetable intake was associated with higher IL-10 levels and lower stress levels in pregnant women with chronic diseases.

## 1 Introduction

Large-scale community studies have shown that 30 %–75 % of pregnant women undergo psychological stress during pregnancy, often triggered by unpleasant events, such as increased conflicts with their partners, family members facing serious illnesses or hospitalizations, and financial struggles (Traylor et al., 2020; Van den Bergh et al., 2020). Psychological stress can present in various ways, including acute stress, chronic stress, and allostatic overload (Traylor et al., 2020). Elevated stress levels during pregnancy have been linked to negative outcomes including preeclampsia, neonatal morbidity, and low birth weight (Bussières et al., 2015; Caparros-Gonzalez et al., 2021; Yu et al., 2013). Women with chronic diseases are particularly susceptible to heightened stress during pregnancy, as they face the dual challenges of managing their pregnancy while also coping with preexisting medical conditions (Merz et al., 2022). This additional disease burden, together with increased worry about pregnancy complications, can exacerbate stress-related emotions such as anxiety and depression (Ralston et al., 2021).

Furthermore, chronic diseases are often associated with inflammation, which can manifest as either acute or chronic (Laveti et al., 2013). Both acute and chronic inflammation serve as adaptive responses with the primary goal of restoring homeostasis in the body (Majumder et al., 2016). While acute inflammation usually lasts 2–6 weeks with mild symptoms such as pain and fever, chronic inflammation can cause inflammatory diseases such as cardiovascular diseases and allergies by disrupting the body's normal homeostatic balance (Kotas and Medzhitov, 2015). Inflammatory cytokines are categorized into two types: pro-inflammatory and anti-inflammatory. Pro-inflammatory cytokines (TNF-α, IL-1, IL-6, IL-1β, etc.) are mainly produced by macrophages and mast cells. They upregulate inflammatory reactions by activating the endothelium, promoting leukocyte infiltration, and triggering the acute phase response. On the other hand, anti-inflammatory cytokines (IL-4, IL-10, IL-11, etc.) counteract inflammation by inhibiting the production of pro-inflammatory cytokines and chemokines in macrophages and dendritic cells, acting as toll-like receptor agonists (Murray, 2005).

Due to concerns over the potential side effects of prolonged pharmaceutical treatments, there is a growing interest in using natural foods as safer alternatives for prevention and management of chronic inflammatory diseases (Majumder et al., 2016). For example, eating lots of vegetables has been shown to offer defense against heart disease, certain cancers, and other chronic diseases by reducing inflammation in the body (Craddock et al., 2019; Haghighatdoost et al., 2017). Vegetables are abundant in immune-boosting compounds such as fiber, folate, vitamins, and various phytochemicals including carotenoids and flavonoids (e.g., β-carotene, anthocyanins, flavanols, and flavanones) (Liu, 2013). These compounds are important for cell growth and the immune system (Hosseini et al., 2018). Additionally, vegetables, high in fiber, can reduce inflammation by affecting gut pH and permeability, which may lead to changes in neurotransmitter levels that could help ease symptoms of depression (Qu et al., 2024; Swann et al., 2019). A vegetable-rich diet also has the potential to modulate feelings of psychological stress (Khaled et al., 2020; Ling and Zahry, 2021; López-Cepero et al., 2021).

In Hong Kong, pregnant women in the second and third trimesters (14th to 40th week) with a normal body mass index and weighing between 40 kg and 60 kg before pregnancy are recommended to consume a minimum of 4–5 servings of vegetables daily (HKSAR Department of Health, 2024a; 2024b). However, a previous study found that 48 % of pregnant women in Hong Kong had inadequate dietary intake, especially with regards to vegetable consumption (Wong et al., 2022). Another study conducted on Japanese pregnant women revealed that insufficient vegetable consumption was associated with frequent dining out, consumption of confectioneries, perceived barriers to obtaining vegetables, lack of awareness of the recommended vegetable intake amounts, and low self-efficacy regarding vegetable intake (Takei et al., 2019).

Vegetables are a key component of an anti-inflammatory diet due to their high polyphenol content, which can reduce inflammation by inhibiting inflammatory mediators and modulating key signaling pathways in the body (Ricker and Haas, 2017). Polyphenols have been found to boost anti-inflammatory cytokines such as interleukin-10 (IL-10) (Yahfoufi et al., 2018). IL-10 acts as a key regulator of the immune system, playing a vital role in maintaining immune response balance, controlling inflammation, and safeguarding tissues from damage. In the event of an infection, IL-10 restrains the function of Th1 cells, NK cells, and macrophages, which are essential for effective pathogen elimination but can also lead to tissue damage (Couper et al., 2008). The reduced levels of IL-10 observed in individuals with chronic inflammatory conditions further highlight the importance of IL-10 in modulating immune responses (Stumpf et al., 2003).

**Fig. 1:**
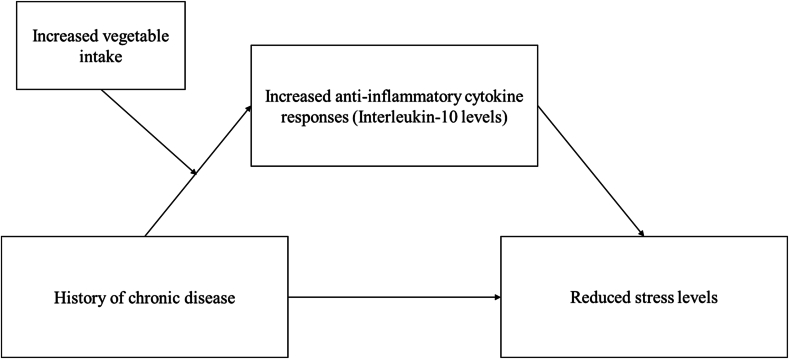
Hypothesized model linking chronic disease, vegetable intake, IL-10 levels, and stress levels.

Given the link between vegetable consumption, anti-inflammatory cytokine IL-10 levels, and psychological stress, vegetable consumption may have protective effects on the mental well-being of individuals with chronic conditions. Hence, this study aimed to examine whether a history of chronic diseases was associated with increased levels of psychological stress in pregnant women and whether anti-inflammatory IL-10 and stress levels differed by vegetable intake among pregnant women with chronic diseases. We hypothesized that (H1) a history of chronic disease was associated with increased stress levels in pregnant women before adjusting for vegetable intake levels. After including vegetable intake in the model, (H2) the association between a history of chronic diseases and anti-inflammatory IL-10 levels would differ by vegetable intake levels. Finally, (H3) the association between chronic disease history and stress levels via anti-inflammatory IL-10 levels was conditional upon vegetable intake, as illustrated in Fig. 1.

## 2 Methods

This is a prospective observational study with institutional ethics approval from the Institutional Review Board of the University of Hong Kong/Hospital Authority Hong Kong West Cluster Research Ethics Committee. Participants provided written informed consent prior to participation in this study. The study group consisted of 239 Chinese expectant mothers attending antenatal clinics in public hospitals. Post-hoc power analysis was conducted using this sample size, which was sufficient to detect a small-to-medium effect size of f2 = 0.065 at a significance level of 0.05 with 80 % power.

### 2.1 Study population

This study recruited 239 pregnant women who could communicate and read Chinese and were between 24 and 28 weeks of gestation during hospital visits from July to October 2019. Exclusion criteria included being under 18 years of age and current smokers. Eligible women were identified in the antenatal clinic by trained staff, who provided an information sheet detailing the study objectives before obtaining their written informed consent. Upon giving written consent to participate, pregnant women provided information on socio-demographics and were asked whether they had a long-term illness or medical condition diagnosed by a doctor (e.g., diabetes, arthritis, heart disease, or allergy). The specific diagnoses provided by the participants were cross-checked with their electronic health records in the hospital database. Additionally, trained phlebotomists collected blood samples for inflammatory marker assays.

## 3 Measures

### 3.1 Vegetable intake assessment

Participants completed vegetable intake assessment on the day of recruitment using an electronic version of a locally validated food frequency questionnaire (eFFQ) (Woo et al., 1997), which has been utilized in previous local research (Wong et al., 2022). The reliability and validity of the Chinese food frequency questionnaire has been extensively established, with correlation coefficients between 0.44 and 0.48 reported for assessing vegetable consumption (Lo et al., 2020; Zhao et al., 2010). Minor modifications were made to remove outdated items and include new food items that are commonly consumed in pregnancy (Yip et al., 2020). The eFFQ contains 311 food items, including 12 food groups: fish and seafood, mushrooms, eggs, dairy beverages, beans, fruits, grains, meats, snacks, soups, vegetables, and condiments and oil. Each participant was asked to fill in the amount and frequency of consumption of each food item in the past month prior to the interview. Frequency options include once a month, 2–3 times per month, once to twice a week, 3–4 times per week, 5–6 times per week, and every day. Common household utensils and containers and pictures of individual food portions were provided to facilitate estimation of portion size. Local dietary guidance (HKSAR Department of Health, 2024a; 2024b) was used to convert intake data to servings of vegetables. In this study, one serving of vegetables is defined as 1 bowl (250–300 mL) raw vegetables or 0.5 bowl cooked vegetables.

### 3.2 Assessment of psychological stress levels

To examine the post-diet effect, one month after the dietary intake assessment, participants were sent a link to complete an electronic version of the Perceived Stress Scale – 10 (PSS-10) (Cohen and Janicki-Deverts, 2012). The scale consists of 10 items that assess a participant's perceived psychological stress levels over the past month. Participants rated each item on a 5-point scale, with responses ranging from 1 = *strongly disagree* to 7 = *strongly agree*. Higher scores indicate higher levels of psychological stress. Example items included questions such as “How often have you been upset because of something that happened unexpectedly?“.

### 3.3 Assessment of anti-inflammatory IL-10

Venous blood samples (9 mL) were collected from participants on the day of recruitment and centrifuged at 3000 revolutions per minute for 15 min. The resulting serum was then transferred into 1 mL tubes for storage at −80 °C. The level of circulating IL-10 was determined by analysis of the supernatants using the LEGENDplex™ Human Inflammation Panel 1 (13-plex) (BioLegend). The assay was carried out in a V-bottom plate following the instructions provided by the manufacturer, and the data was collected using the Flow Cytometer—Cytoflex S with High Throughput System from Beckham Coulter. Analysis was conducted using BioLegend's LEGENDplex Data Analysis Software available at www.biolegend.com/legendplex.

### 3.4 Covariates

All covariates were selected a priori based on past research. Demographic covariates included maternal age (in years) and monthly household income, while biomedical covariates included gravida (number of pregnancy) and pre-pregnancy body mass index (BMI) (Fantuzzi, 2005). Pre-pregnancy BMI (kg/m2) was calculated using maternal self-reported height (cm) and weight (kg). Gestational age at enrollment (in weeks) was also included to control for the effect of pregnancy duration.

### 3.5 Data analysis

We applied the SPSS PROCESS macro to evaluate the hypothesized moderated mediation model. This macro examines direct, indirect (i.e., mediation), and interaction (i.e., moderation) effects through bias-corrected bootstrapping within a regression framework. Specifically, it uses random sampling with replacement to generate average indirect effects from 5000 bootstrap samples. It also calculates and examines interactions to determine whether the direct or indirect effects depend on varying levels of the moderator (i.e., vegetable intake in this study). A statistically significant interaction term suggested that vegetable intake emerged as a moderator of the indirect effects. Moreover, this macro offers a specific index of moderated mediation. A statistically significant index of moderated mediation indicates the presence of a true moderated mediation effect. This means that the indirect association between chronic disease history and psychological stress via the mediating variable (IL-10 levels) was dependent on vegetable intake.

## 4 Results

Among the 239 participants, no missing data was found. To address data skewness, IL-10 levels were log10-transformed. Descriptive statistics including the means and SDs of study variables for the overall sample and stratified by a history of chronic diseases are presented in Table 1. The ages of participants ranged from 23 to 38 (mean age 32.54, SD = 3.32). On average, the participants were in their 26th week of pregnancy and consumed four servings of vegetables per day, meeting the locally recommended daily vegetable intake for pregnant women during the second and third trimesters (HKSAR Department of Health, 2024b). Additionally, 15 % (n = 37) self-reported being diagnosed with chronic diseases, including lung disease (n = 1), kidney disease (n = 1), liver disease (n = 8), autoimmune disease (n = 19), thalassemia (n = 5), and allergies (n = 3). Participants without chronic diseases weighed significantly more than those with chronic diseases before pregnancy (p = 0.033), but there were no significant differences in the number of vegetable servings and levels of circulating IL-10 between participants with chronic diseases and those without. Notably, pregnant women with chronic diseases reported significantly higher levels of psychological stress compared to those without chronic diseases (p = 0.003), providing support for our first hypothesis (H1). Table 2 presents the Pearson correlations between study variables. There was a significant association between vegetable intake and IL-10 levels in participants with chronic diseases, but not in those without. On the other hand, psychological stress levels were significantly associated with IL-10 levels in participants without chronic diseases, but not in those with chronic diseases.

**Table 1:** Descriptive statistics.

	Overall (n = 239)	With chronic disease (n = 37)	Without chronic disease (n = 202)	p-value
	mean (SD)	mean (SD)	mean (SD)	
Maternal age	32.54(3.32)	32.30(3.63)	32.58(3.27)	0.636
Monthly household income (USD '000)	3.30(2.17)	3.20(2.07)	3.31(2.19)	0.883
Gestational age	26.38(2.12)	26.38(2.11)	26.39(2.13)	0.984
Pre-pregnancy BMI	22.40(3.10)	21.41(3.19)	22.59(3.06)	0.033
Log10-transformed IL-10	0.72(0.41)	0.74(0.40)	0.72(0.41)	0.755
Vegetable intake	4.02(2.51)	4.01(2.30)	4.03(2.56)	0.967
Stress	16.04(4.44)	18.59(5.65)	15.57(4.02)	0.003
Gravida	1.23(0.55)	1.30(0.62)	1.21(0.54)	0.391

**Table 2:** Correlational statistics.

		1	2	3	4	5	6	7	8
Overall (n = 239)	1 Log10-transformed IL-10	–	–	–	–	–	–	–	–
	2 Vegetable intake	0.06	–	–	–	–	–	–	–
	3 Stress	−0.15^a^	−0.09	–	–	–	–	–	–
	4 Gravida	−0.02	−0.07	0.11	–	–	–	–	–
	5 Maternal age	−0.05	−0.10	−0.10	0.17∗∗	–	–	–	–
	6 Monthly household income (USD '000)	0.002	0.10	−0.03	0.02	0.14^a^	–	–	–
	7 Gestational age	0.04	−0.09	−0.02	0.02	0.09	0.07	–	–
	8 Pre-pregnancy BMI	0.01	0.05	−0.07	0.03	0.05	−0.16^a^	−0.10	–
With chronic disease (n = 37)	1 Log10-transformed IL-10	–	–	–	–	–	–	–	–
	2 Vegetable intake	0.48∗∗	–	–	–	–	–	–	–
	3 Stress	−0.04	−0.22	–	–	–	–	–	–
	4 Gravida	−0.16	−0.31	0.43^a^	–	–	–	–	–
	5 Maternal age	−0.12	−0.15	−0.35^a^	0.23	–	–	–	–
	6 Monthly household income (USD '000)	−0.17	0.004	−0.32	0.02	0.14	–	–	–
	7 Gestational age	0.14	0.01	−0.09	−0.07	0.18	0.10	–	–
	8 Pre-pregnancy BMI	−0.19	−0.38^a^	0.31	0.28	0.28	−0.31	0.13	–
Without chronic disease (n = 202)	1 Log10-transformed IL-10	–	–	–	–	–	–	–	–
	2 Vegetable intake	−0.01	–	–	–	–	–	–	–
	3 Stress	−0.19∗∗	−0.07	–	–	–	–	–	–
	4 Gravida	0.01	−0.03	0.01	–	–	–	–	–
	5 Maternal age	−0.03	−0.09	−0.03	0.16^a^	–	–	–	–
	6 Monthly household income(USD '000)	0.03	0.11	0.04	0.02	0.14	–	–	–
	7 Gestational age	0.02	−0.11	−0.01	0.03	0.07	0.06	–	–
	8 Pre-pregnancy BMI	0.05	0.12	−0.12	−0.01	−0.002	−0.14^a^	−0.14^a^	–

a p < 0.05, ∗∗p < 0.01.

**Fig. 2:**
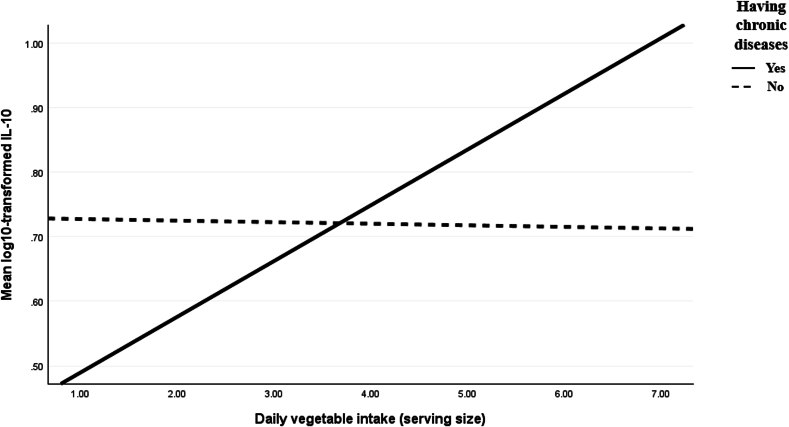
Mean IL-10 levels by chronic disease history across vegetable intake levels.

Next, we explored whether the association of chronic disease history with psychological stress through IL-10 levels was conditional on the amount of vegetable intake. Table 3 reports the results of moderated mediation analyses for the indirect association of chronic disease history with psychological stress via IL-10 levels at different levels of vegetable consumption. The results indicated that the interaction between chronic disease history and vegetable intake significantly predicted IL-10 levels (B = 0.09, t = 2.72, p = 0.007), providing full support for our second hypothesis (H2). Fig. 2 presents the simple slopes plot illustrating how the relationship between chronic disease history and IL-10 levels changed across different levels of vegetable intake. Higher levels of vegetable intake were associated with higher IL-10 levels only in pregnant women with chronic diseases (B = 0.07, p = 0.015). The association between chronic disease history and psychological stress via IL-10 levels also differed by vegetable intake (index of moderated mediation: −0.15; Boot SE = 0.09; LLCI = −0.36; ULCI = −0.01), thus supporting our third hypothesis (H3). Specifically, a history of chronic diseases was associated with reduced psychological stress via increased IL-10 only at high levels of vegetable intake (B = −0.43; Boot SE = 0.28; LLCI = −1.06; ULCI = −0.01), as zero was not included in the CI level.

**Table 3:** Testing the moderated mediation effect of chronic diseases and vegetable intake on IL-10 and psychological stress among pregnant women.

Outcome	Predictors	R^2^	F	B	LLCI	ULCI	t value
log10-transformed IL-10	Gravida	0.04	1.16	0.01	−0.09	0.10	0.13
	Age			−0.01	−0.02	0.01	−0.68
	Family income			0.002	−0.02	0.02	0.23
	Gestational age			0.01	−0.02	0.03	0.66
	Pre-pregnancy BMI			0.01	−0.01	0.02	0.74
	Vegetable intake			−0.002	−0.03	0.02	−0.21
	Chronic disease			−0.33	−0.62	−0.03	−2.19∗
	Disease x Vegetable			0.09	0.02	0.15	2.72∗∗
Psychological stress	Gravida	0.11	4.11	0.95	−0.06	1.95	1.86
	Age			−0.16	−0.33	0.01	−1.87
	Family income			−0.03	−0.23	0.17	−0.31
	Gestational age			−0.02	−0.28	0.24	−0.17
	Pre-pregnancy BMI			−0.05	−0.23	0.13	−0.53
	Log10 transformed IL-10			−1.72	−3.05	−0.40	−2.56∗
	Chronic disease			2.88	1.36	4.39	3.74∗∗∗
Conditional effect of chronic disease on log10-transformed IL-10 at the values of the moderator:				B	Boot SE	BootLLCI	BootULCI
1.70 serving per day				−0.18	0.10	−0.38	0.03
3.37 servings per day				−0.03	0.08	−0.18	0.12
6.46 servings per day				0.25	0.11	0.03	0.46
Conditional indirect effect analysis at the values of the moderator:				B	Boot SE	BootLLCI	BootULCI
1.70 serving per day				0.30	0.23	−0.02	0.81
3.37 servings per day				0.05	0.13	−0.19	0.33
6.46 servings per day				−0.43	0.28	−1.16	−0.01

Note: N = 239, logIL-10 = log10-transformed IL-10, BMI = body mass index. Bootstrap sample size = 5000, LL = low limit, CI = confidence interval, UL = upper limit. The beta coefficient was unstandardized. ∗p < 0.05, ∗∗p < 0.01, ∗∗∗p < 0.001.

## 5 Discussion

Our study is the first to test whether women with chronic diseases experienced higher levels of psychological stress during pregnancy compared to those without such conditions. We found that pregnant women with chronic conditions and low vegetable intake experienced higher stress levels. However, when these women consumed high levels of vegetables, they showed higher levels of the anti-inflammatory cytokines IL-10 and lower psychological stress levels compared to those without chronic conditions. Elevated levels of psychological stress can have detrimental effects on the health of both the fetus and the mother (Traylor et al., 2020). Pregnant women with chronic diseases may exhibit increased signs of inflammation compared to their counterparts without such conditions, potentially placing them at an elevated level of psychological stress. However, studies also suggested that vegetable consumption could help combat chronic inflammation and stress in the body (Khaled et al., 2020; Ling and Zahry, 2021; López-Cepero et al., 2021).

Our first hypothesis, suggesting that a history of chronic disease was associated with increased stress levels in pregnant women before adjusting for vegetable intake levels, was supported. We found that pregnant women with chronic diseases reported higher psychological stress levels compared to those without such conditions. This finding is consistent with previous research that links perceived stress with chronic diseases (Richardson et al., 2012; Tenk et al., 2018). The interconnectedness of chronic diseases and stress can be explained in two ways. Firstly, viewing stress as a causative factor, stress has the potential to trigger heightened inflammation, elevate blood pressure, compromise the immune system, and disrupt hormonal balance, all recognized as risk factors for chronic diseases (Richardson et al., 2012; Tenk et al., 2018). Secondly, from the perspective of symptoms inducing stress, individuals with chronic diseases may experience physical discomfort, social isolation, and other factors that can exacerbate their stress levels (Fayaz et al., 2016; Mellado et al., 2016). Our study builds upon these insights by highlighting the varying levels of stress associated with chronic conditions in pregnant women, underscoring the importance of tailored mental health support for pregnant women dealing with chronic diseases.

Our second hypothesis, which proposed that the association between a history of chronic diseases and anti-inflammatory IL-10 levels differed by vegetable intake levels, was also confirmed. Consistent with previous reports of notable variations in vegetable consumption among pregnant women (Wong et al., 2022), this study also found a substantial number of participants with low vegetable intake. If analyzing the overall sample without accounting for vegetable intake variations, the benefits of adequate vegetable consumption could have been obscured. Therefore, we employed a moderated mediation model to capture the unique anti-inflammatory response patterns that define the small subset of participants with high vegetable consumption levels. Specifically, prior to considering the effect of vegetable intake, a history of chronic diseases was associated with reduced IL-10 levels. Furthermore, pregnant women with chronic conditions experienced heightened psychological stress. Nonetheless, following the inclusion of vegetable consumption levels in the model, we observed shifts in both IL-10 levels and stress patterns, underscoring the importance of vegetable intake. These results align with the notion that psychological stress was associated with an increase in both pro- and anti-inflammatory cytokines (Steptoe et al., 2007; Szabo et al., 2020). The regulation of these cytokines is crucial for managing stress responses internally. In healthy individuals, the body can effectively restore a state of equilibrium, known as homeostasis, to sustain survival and normal functions. However, individuals with chronic diseases may have a compromised capacity to maintain homeostasis, rendering them vulnerable to inflammation (Kotas and Medzhitov, 2015).

The third hypothesis, which proposed that the association between chronic disease history and stress levels via anti-inflammatory IL-10 levels was conditional upon vegetable intake, was supported. We observed that higher IL-10 levels resulting from increased vegetable consumption was associated with lower stress levels in pregnant women with chronic diseases. This finding further emphasizes the mental health benefits of an anti-inflammatory diet, as noted in previous research (Jiang et al., 2022; Lv et al., 2022). Vegetables, being a fundamental element of an anti-inflammatory diet, possess immune-boosting properties (Ricker and Haas, 2017). Previous studies have demonstrated that adhering to anti-inflammatory diets can enhance mental well-being, whereas diets rich in inflammatory components are associated with an increased risk of depression (Jiang et al., 2022; Lv et al., 2022). Our research expands the current literature by demonstrating the benefits of vegetable consumption to boosting anti-inflammatory cytokine IL-10 levels in pregnant women with chronic diseases. Specifically, by consuming vegetables at or above the recommended levels (i.e., 4–5 servings per day), pregnant women with chronic diseases showed similar or even higher levels of anti-inflammatory responses compared to those without such conditions. This observation is consistent with evolutionary theories, such as the biological sensitivity to context theory and the differential susceptibility theory (Ellis et al., 2011), which suggest that individuals who are most susceptible to negative environmental conditions may benefit the most from positive influences. Pregnant women with chronic diseases could be more likely to get sick, yet it is plausible that, due to this susceptibility, they could have increased receptivity to a vegetable-based diet, as illustrated by their elevated anti-inflammatory cytokine levels observed in this study. While previous research has applied these theories to explore individual differences in environmental susceptibility, our findings further highlight a new possibility of investigating susceptibility to dietary influences in a “for better and for worse” manner.

### 5.1 Clinical implications

The clinical implications of this study suggest that increasing vegetable intake during pregnancy may have beneficial effects on inflammation and mental health, especially in women with chronic diseases. Encouraging higher vegetable consumption could help elevate anti-inflammatory cytokine IL-10 levels, which are associated with reduced psychological stress, potentially improving overall maternal well-being and pregnancy outcomes. For pregnant women with chronic conditions, dietary strategies that include vegetables might serve as a complementary approach to traditional treatments by reducing inflammation and stress-related complications. Additionally, the association between elevated IL-10 levels and reduced stress highlights the potential mental health benefits of a vegetable-rich diet during pregnancy. Based on these observations, further research is needed to explore disease-specific mechanisms and to replicate the findings across diverse populations, ensuring that dietary recommendations are inclusive and effective on a broader public health scale. Overall, these findings support integrating dietary guidance focusing on vegetable consumption into prenatal care, particularly for women with chronic health conditions, to promote better inflammatory and psychological outcomes during pregnancy.

### 5.2 Limitations

This study has several limitations. Firstly, although the present study offers a novel perspective by sampling pregnant women both with and without chronic diseases, it is important to note that each chronic disease may have its own specific mechanism. Future research should explore disease-specific mechanisms by recruiting a larger sample focused on a particular disease of interest. Collecting more diagnositic information, such as disease onset time and severity and medication use, would also be beneficial for a more comprehensive analysis. Secondly, the present study focused on pregnant women. Testing the present model in more diverse populations is essential to validate the significance of vegetable consumption in enhnacing anti-inflammatory responses for stress management. Thirdly, the use of subjective dietary intake assessment methods may introduce recall bias. Future research should include objective measures and consider additional lifestyle factors beyond dietary patterns, such as sleep patterns and physical activity habits. Lastly, the psychological stress level was assessed using the Perceived Stress Scale, which is not an objective marker of stress. Combining the subjective (Perceived Stress Scale) and objective stress markers (such as cortisol or catecholamines) would have improved the reliability of the findings.

## 6 Conclusions

This study demonstrates a significant association between vegetable intake and anti-inflammatory cytokine IL-10 levels in pregnant women with chronic diseases. Higher IL-10 levels are associated with reduced psychological stress. The consumption of vegetables is particularly beneficial for pregnant women with chronic diseases, as shown by elevated IL-10 levels in those with high vegetable consumption. Future research should explore disease-specific mechanisms in relation to inflammation markers and stress-related emotions during pregnancy. It is also essential to replicate these findings in diverse populations to confirm the mental health benefits of vegetable-rich diets on a broader public health scale.

## CRediT authorship contribution statement

**Rosa S. Wong:** Writing – review & editing, Writing – original draft, Methodology, Investigation, Funding acquisition, Formal analysis, Data curation, Conceptualization. **Keith T.S. Tung:** Writing – review & editing, Methodology, Investigation, Data curation, Conceptualization. **Patrick Ip:** Writing – review & editing, Methodology, Investigation, Data curation, Conceptualization.

## Availability of data and material

The data that support the findings of this study are available on request from the corresponding author.

## Funding

This study was funded by the start-up research grant for newly recruited academic staff (RG 20/2023-2024R) from The 10.13039/501100010410Education University of Hong Kong.

## Financial disclosure

All authors have indicated they have no financial relationships relevant to this article to disclose.

## Declaration of competing interest

All authors have indicated they have no potential conflicts of interest to disclose.

## Data Availability

Data will be made available on request.
